# Hydroquinone shows neuroprotective potential in an experimental ischemic stroke model via attenuation of blood-brain barrier disruption

**DOI:** 10.1186/cc14542

**Published:** 2015-03-16

**Authors:** JH Cho, CW Park, TG Ohk, MC Shin, MH Won

**Affiliations:** 1 Kangwon National Univeristy, Chuncheon-si, Gangwondo, South Korea

## Introduction

Hydroquinone (HQ), a major benzene metabolite, occurs naturally in various plants and food, and is also manufactured for commercial use. Although many studies have demonstrated the various biological effects of HQ, the neuroprotective effects of HQ following ischemic stroke have not been investigated. Therefore, in this study, we first examined the neuroprotective effects of HQ against ischemic damage in a focal cerebral ischemia rat model.

## Methods

It was proven that pre and post treatment with 100 mg/ kg HQ protects from ischemia-induced cerebral damage, which was confirmed by evaluation of neurological deficit, positron-emission tomography and 2,3,5-triphenyltetrazoliumchloride staining.

## Results

In addition, pre and post treatment with 100 mg/kg HQ significantly attenuated ischemia-induced Evans blue dye extravasation, and significantly increased the immunoreactivities and protein levels of SMI-71 and glucose transporter-1 (GLUT-1), which were well known as useful makers of endothelial cells, in ischemic cortex compared with a vehicle-treated group.

## Conclusion

Briefly, these results indicate that pre and post treatment with HQ can protect from ischemic damage induced by transient focal cerebral ischemia, and the neuroprotective effects of HQ may be closely associated with the prevention of BBB disruption via increase of SMI-71 and GLUT-1 expressions.

